# Intrinsic links among sex, emotion, and reproduction

**DOI:** 10.1007/s00018-018-2802-3

**Published:** 2018-03-26

**Authors:** Lisa Yang, Alexander N. Comninos, Waljit S. Dhillo

**Affiliations:** 1 Section of Endocrinology and Investigative Medicine, Imperial College London, 6th Floor, Commonwealth Building, Hammersmith Hospital Campus, Du Cane Road, London, W12 ONN UK; 2 Department of Endocrinology, Imperial College Healthcare NHS Trust, London, UK

**Keywords:** Amygdala, Cortisol, Emotion, Endocrine, Hypothalamus, Kisspeptin, Limbic system, Psychosexual, Reproduction, Sexual behaviour

## Abstract

Species survival is dependent on successful reproduction. This begins with a desire to mate, followed by selection of a partner, copulation and in monogamous mammals including humans, requires emotions and behaviours necessary to maintain partner bonds for the benefit of rearing young. Hormones are integral to all of these stages and not only mediate physiological and endocrine processes involved in reproduction, but also act as neuromodulators within limbic brain centres to facilitate the expression of innate emotions and behaviours required for reproduction. A significant body of work is unravelling the roles of several key hormones in the modulation of mood states and sexual behaviours; however, a full understanding of the integration of these intrinsic links among sexual and emotional brain circuits still eludes us. This review summarises the evidence to date and postulates future directions to identify potential psycho-neuroendocrine frameworks linking sexual and emotional brain processes with reproduction.

## Introduction

Reproduction is an essential physiological process for preservation of the species, which relies on the integration of social and sensory cues with emotional and behavioural outputs that favour the successful production of offspring. Effective mating depends on an appropriate display of sexual behaviours that select for reproductive fitness. The neural command centre for coordinating these complex processes sits within the limbic brain [1]. The limbic system was first described by Thomas Willis in 1664 and is one of the most studied functional networks in the brain [1]. Evolutionarily preserved since amphibia and common among all mammals, it consists of several functionally interconnected cortical and subcortical structures that have a shared role in processing sensory stimuli into emotional and behavioural outputs [2]. The currently accepted composition of the limbic system is derived from Paul MacLean’s proposal in 1952. This includes the orbitofrontal cortex, hippocampus, cingulate cortex, amygdala, hypothalamus, thalamus, and the ventral striatum (i.e., nucleus accumbens) [1]. Within the limbic network, several structures are thought to be predominantly involved in processing emotions; these include the amygdala, hypothalamus, cingulate cortex, and pre-frontal cortex [3, 4].

The amygdala is a small almond-shaped structure lying deep to the antero-inferior temporal lobe. Animal lesion studies have established the amygdala as a central hub for processing emotions and sexual behaviours in rodents and primates [3, 5, 6]. In humans, there is a positive association between sexual drive and amygdala volume [4]. Functional magnetic resonance imaging (fMRI) demonstrates enhanced amygdala activity in response to viewing sexually arousing images in men and women [7]. The medial nucleus of the amygdala is particularly implicated in processing multimodal sensory inputs such as olfactory signals and integrating these inputs into behavioural and endocrine pathways [8, 9].

The hypothalamus coordinates essential homeostatic mechanisms including the regulation of neuroendocrine axes and autonomic processes. Limbic functions of the hypothalamus have been demonstrated in rats, whereby hypothalamic stimulation leads to increased motivation towards rewarding behaviours [10]. The amygdala and hypothalamus are highly interconnected and electrical stimulation of the amygdala in rats and cats leads to increased gonadotropin secretion [8, 11]. This demonstrates that the amygdala–hypothalamus connection serves as a conduit between emotion-processing brain centres and reproductive neuroendocrine pathways.

The hypothalamic–pituitary–gonadal (HPG) axis orchestrates physiological processes required for reproduction. Furthermore, it is known that hypogonadal states in animals and humans are associated with loss of responsiveness to sexual stimuli and loss of appetitive sexual behaviours [12–14]. Reproductive hormones and their receptors are found throughout the limbic brain network where evidence suggests that they act as neurotransmitters and neuromodulators to influence emotional states and sexual behaviour [15]. Thus, a considerable interest has developed with regard to factors that may integrate reproductive endocrinology with sexual behaviours. Recently, the discovery of novel neuropeptides acting above the level of gonadotropin-releasing hormone (GnRH) to modulate its release have redefined our understanding of reproductive endocrinology and also provide new candidates for the study of intrinsic links between reproduction, emotion, and sexual behaviours.

This review summarises the literature surrounding key hormones and their roles in sexual and emotional brain processing. We examine common functions and discuss future directions for elucidating the psycho-neuroendocrine links uniting sex, emotion, and reproduction.

## Kisspeptin

**Fig. 1:**
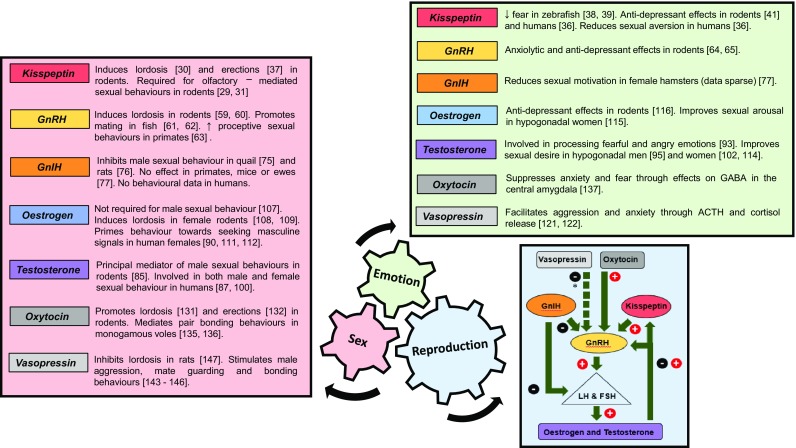
Summary of the effects of key hormones in the control of sex, emotion, and reproduction. Relevant references in parentheses. *ACTH* adrenocortico-trophic hormone, *GABA* gamma-aminobutyric acid, *GnRH* gonadotropin-releasing hormone, *GnIH* gonadotropin-inhibiting hormone. * Vasopressin has indirect inhibitory effects on GnRH through stimulation of the hypothalamic–pituitary–adrenal axis

Kisspeptin refers to a family of peptide hormones cleaved from the product of the Kiss1 gene. They share a common carboxyl terminal sequence obligate for their action on kisspeptin receptors (encoded by Kiss1r) [16]. Kisspeptin is secreted by kisspeptin neurones within the hypothalamus and activates kisspeptin receptors located on GnRH neurones to stimulate GnRH release and downstream reproductive hormone secretion (Fig. 1). The absence of Kiss1/Kiss1r results in a failure to go through puberty with resultant infertility [17, 18]. Conversely, activating mutations of Kiss1/Kiss1r trigger central precocious puberty [19]. These findings demonstrate the crucial importance of kisspeptin as a potent activator of the HPG axis, although the extent to which activation occurs can vary according to various other factors such as nutrition [20] and season [21]. In seasonally breeding hamsters, reproductively in-active short day females display significantly increased LH levels following a 5 uM injection of kisspeptin, whereas reproductively active long day females show no significant elevation in LH [21]. Recent data also suggest an important role for kisspeptin in the modulation of sexual and emotional brain processing.

### Distribution of kisspeptin in the limbic system

Kisspeptin-expressing neurones and mRNA have been identified in the bed nucleus of the stria terminalis (BNST), thalamus, and amygdala of rodents [22]. Furthermore, amygdala kisspeptin expression is positively modulated by gonadal sex steroids [23]. Kisspeptin receptor expression has also been demonstrated in rodent limbic brain structures, including the amygdala, thalamus, hippocampus, and olfactory systems [24–26]. Of note, direct kisspeptin administration into the medial amygdala stimulates LH secretion, while direct kisspeptin antagonist administration decreases LH secretion and pulsatility, providing evidence for a link between amygdala kisspeptin signalling and the HPG axis [27]. In humans, KISS1 and KISS1R mRNA have been identified in several limbic and paralimbic structures including the amygdala, caudate, cingulate, globus pallidus, hippocampus, medial and superior frontal gyrus, nucleus accumbens, parahippocampal gyrus, substantia nigra, putamen, and thalamus [28, 29]. Thus, there exists an anatomical framework in place through which kisspeptin signalling can link reproduction, sex, and emotion.

### Kisspeptin’s role in sex and emotion

The first evidence for kisspeptin’s role in sexual behaviour came from Keller et al., who show that peripheral administration of kisspeptin stimulates the lordosis reflex in female mice [30]. Later, Kauffman and colleagues demonstrate that testosterone-replaced Kiss1r knock-out male mice lack olfactory partner preference despite normosmia [31]. In keeping with this, activation of medial amygdala kisspeptin neurones increases time spent by male mice investigating females [32]. Furthermore, studies demonstrate that opposite sex odours activate kisspeptin neurones in rodents [33, 34]. This effect is also seen in sheep, whereby kisspeptin cFos activity is increased after exposure of anoestrous ewes to a novel male with an associated rise in LH pulsatility [35]. In healthy young men, kisspeptin enhances limbic and paralimbic brain activity on fMRI, specifically in response to sexually arousing and non-sexual couple-bonding images [36]. In addition, kisspeptin enhancement of brain activity (in response to sexual images) in several of these structures correlates with decreased sexual aversion [36]. In keeping with this human data, direct kisspeptin injection into the medial amygdala dose-dependently triggers multiple erections in male rats, but is blocked by pre-treatment with a kisspeptin antagonist [37].

Data also suggest a role for kisspeptin in the display of fear and anxiety. In zebrafish, fear stimuli significantly reduce Kiss1 transcription, while intra-cranial kisspeptin administration attenuates fear responses via effects on the serotonergic system [38, 39]. In terms of function, kisspeptin administration may increase anxiety [40] or decrease anxiety [32] depending on the methodology used. Kisspeptin administration has also been shown to have anti-depressant-like effects in rodents [41] and humans [36] with the latter effect associated with enhanced activity in the pre-frontal area, a region that expresses kisspeptin receptor mRNA in humans and known to be involved in negative emotions [28].

Mechanistically, there is observed interplay between kisspeptin signalling and several other neuropeptide systems including serotoninergic [39], adrenergic [41], vasopressinergic [26], dopaminergic [26], nitric oxide [42], gamma-aminobutyric acid (GABA), glutamate [43], and CART systems [44]. In addition, data suggest that, when peripherally administered, certain isoforms of kisspeptin can cross the blood–brain barrier to reach its central receptors located throughout the brain including in the limbic system as above [36, 45].

### Summary

Collectively, these studies in humans and several other species demonstrate that kisspeptin signalling effectively integrates sensory processing with limbic pathways involved in sexual arousal, positive mood, and anxiety (for a current review of kisspeptin in these areas, please see [46]). Together with its established role as a modulator of reproductive hormone secretion, these data suggest that kisspeptin plays a key part in the integration of reproduction, sex, and emotion (Fig. 1), which may be of particular clinical interest for the development of kisspeptin-based therapies.

## Gonadotropin-releasing hormone

GnRH is secreted in a pulsatile fashion from the pre-optic area of the hypothalamus. GnRH binds to its receptor on the anterior pituitary to stimulate LH and follicle-stimulating hormone (FSH) release, which induce sex steroid secretion and gametogenesis in the gonads. Once thought to be the master regulator of the HPG axis, it is now known that GnRH release is controlled by two novel neuropeptides; kisspeptin which exerts a stimulatory effect on GnRH [47] and gonadotropin-inhibitory hormone (GnIH) which exerts a negative effect on GnRH signalling [48] (Fig. 1).

### GnRH in the limbic system

Anatomical localisation of GnRH-expressing neurones has been studied in many species [49–51] with results in rats showing extensive extra-hypothalamic distribution, particularly in the olfactory bulb and tubercle, BNST, hippocampus, and medial amygdala [51]. The GnRH receptor has been immunohistochemically located in several extra-hypothalamic brain areas including the amygdala, hippocampus, piriform (olfactory) cortex, arcuate nucleus, and olfactory bulb in mice [52, 53]. This pattern of distribution for GnRH and its receptor provides an anatomical framework for GnRH to relay olfactory signals between emotion and reproductive centres in the brain. Male hamsters are particularly reliant on olfactory cues to induce sexual behaviour, which they sense via the vomeronasal organ (VNO). Removal of the VNO results in loss of mating behaviour in sexually naïve males, which can be restored by administration of GnRH [54]. Furthermore, this return of sexual behaviour is accompanied by increased cFos activity in the anteroventral medial amygdala [54]. In humans, two studies utilising similar techniques of computer-assisted microscopy to map the distribution of GnRH-expressing neurones [55] and GnRH gene transcripts [56] have demonstrated the presence of GnRH neurones in the anterior olfactory areas, cortical and medial amygdala, and the BNST. However, the functional GnRH receptor appears to be restricted to the pituitary and hippocampus in humans [57] with limited descriptions of its presence in specific limbic brain regions [58].

### GnRH’s role in sex and emotion

In 1973, two independent research groups showed that subcutaneous administration of GnRH to ovariectomised, steroid-replaced female rats potentiates female sexual behaviour, i.e., the lordosis response [59, 60]. Furthermore, bilateral lesions of the medial amygdala lead to reduced cFos expression in GnRH neurones with associated inhibition of lordosis behaviour in female rats [9], demonstrating that GnRH is a neuroendocrine mediator of limbic brain processing in rodents. Since then, GnRH activity has been shown to facilitate a range of sexual behaviours in other species, ranging from mating preferences in fish [61, 62] to proceptive (sexual advancement) behaviours in marmoset monkeys [63].

GnRH agonists are associated with anxiolytic effects in rodent models [64], whereas GnRH antagonists induce anxiogenic behaviours [65]. GnRH also interacts with other neuropeptides and neurotransmitters such as vasopressin [66], GABA [67], and dopamine [68], which may play interconnecting roles in its regulation of mood and behaviours.

### Summary

GnRH and its receptor are distributed in key brain areas involved in processing olfactory cues and integrating chemosensory information with appropriate sexual behaviours in animals (Fig. 1). By contrast, detailed neuroanatomical localisation of the GnRH receptor is limited to the pituitary and hippocampus in humans. GnRH is also involved in ameliorating anxiety which complements its role in facilitating successful reproduction. GnRH is known to regulate and be regulated by several other neurotransmitters; however, the interplay between these factors in the control of emotions and sexual behaviours is complex and not yet fully understood.

## Gonadotropin-inhibitory hormone

GnIH belongs to the RF amide family of peptides designated by their C-terminal arginine (R) and amidated phenylalanine (F) residues. First identified in the Japanese quail, GnIH is now known to be a key regulator of reproduction in avian species and has also been found in other vertebrates [48]. Two active human homologs of GnIH have been immunohistochemically identified using avian GnIH. These are named RF amide-related peptide-1 (RFRP-1) and RFRP-3 [69]. GnIH acts on its cognate receptor GPR147 to exert an inhibitory effect on GnRH neurones in the pre-optic area (Fig. 1) [48]. GnIH neurones also project to the median eminence to directly inhibit LH release (Fig. 1) [70]. Data in humans show that GnIH is able to inhibit LH secretion in post-menopausal women but does not affect kisspeptin-stimulated LH secretion in normal men [71], suggesting that GnIH is unable to overcome the positive stimulus of kisspeptin on the human HPG axis.

### GnIH in the limbic system

GnIH immunoreactive fibres have been identified in brain regions outside the hypothalamus in quail [72], rats [73], and rhesus macaques [74], but this has not yet been examined in humans. Differences in distribution are seen between rodents and primates, with GnIH immunoreactivity present in the amygdala of rats but not primates [73, 74]. However, GnIH immunoreactive fibres do extend to dopaminergic neurones in the ventral tegmental area in primate brains [74] which eludes to a potential role in reward and motivation. As a relatively novel neuropeptide, with fewer published studies than its counterparts, detailed neuroanatomical studies of the GnIH receptor GPR147, are sparse with work mostly concentrated in avian species. Ukena et al. used Southern hybridisation in the Japanese quail to demonstrate expression of GPR147 in the cerebrum, diencephalon, mesencephalon, and spinal cord [72], but more detailed localisation to specific limbic structures has not yet been performed.

### GnIH’s role in sex and emotion

Alongside its inhibitory role on the HPG axis, there is also evidence that GnIH has a negative impact on male sexual behaviours in quail [75] and rats [76] and reduced sexual motivation in female hamsters [77]. However, there may be species differences as another study showed no effect of GnIH on sexual behaviour in cynomolgus monkeys, mice, and ewes [78]. Differences in methodologies and the reproductive status of the animals may have also contributed to these findings.

### Summary

Through its downregulation of the HPG axis and inhibition of sexual behaviours, GnIH may act as a reproductive brake by appropriately inhibiting mating during unfavourable times such as stressful situations (Fig. 1). This is of particular importance in seasonal breeders such as birds; however, it is uncertain whether GnIH significantly affects sexual behaviour consistently in other species.

## Gonadal steroids

Exposure to gonadal steroids such as oestrogen and testosterone during critical developmental windows not only determines sexually dimorphic structural differences, but also influences sex-specific behaviours in many species including humans [79]. Oestrogen and testosterone are essential for physiological neuroendocrine processes and behaviours necessary for sexual maturation and successful copulation.

### Distribution of oestrogen and testosterone in the limbic system

Oestrogen receptors (ERs) and testosterone-binding androgen receptors (ARs) are intra-cellular receptors found throughout the brains of rodents [80, 81]. Using in situ hybridisation in male and female rats, Sar et al. found high densities of ER and AR mRNA in neuronal populations within key hypothalamic regions known to mediate copulatory behaviours, and in limbic regions providing strong inputs to the hypothalamus such as the medial amygdala and the BNST [80]. AR mRNA is also found in brainstem structures involved with sensory function and regions containing somatic motor neurones. Sex differences in AR and ER mRNA distribution are found in the hypothalamus where male rats have a larger pre-optic nucleus containing greater AR mRNA-labelled cells compared to females, whereas the AVPV is twice as large in female rats with significantly more cells expressing ERs compared to males [80, 82]. In the cortex and brainstem, there are far fewer ER labelled cells compared to AR labelled cells in both males and females [81]. Both AR and ER mRNA are found in the olfactory bulb and the olfactory tubercle of male and female rats with AR more densely distributed than ER, particularly in the mitral cell layer [80], which is in keeping with evidence that gonadal steroids have important roles in olfactory signalling for the regulation of social behaviours in rodents as well as other species [83].

### Testosterone’s role in sex and emotion

Testosterone was first found to be critical for male sexual behaviour in 1849, when Berthold showed that castrated roosters lost their secondary sexual characteristics and failed to display copulatory behaviours such as vocalisation and aggression. Furthermore, these behaviours were restored by transplantation of functioning testes from another animal [84]. Subsequent studies using anti-androgens have supported and developed these findings. In testosterone-replaced gonadectomised male mice, blocking AR with systemic administration of the anti-androgen, hydroxyflutamide prevents the restoration of male sexual behaviour (partner preference, scent marking, and ultrasonic vocalisations) whereas blockade of ER does not fully inhibit these behaviours [85]. Intracranial implants of hydroxyflutamide into the medial pre-optic area and ventromedial hypothalamus successfully prevent restoration of sexual behaviour in gonadectomised testosterone-replaced male mice, whereas hydroxyflutamide in the medial amygdala only has partial effects, and no effects when implanted into the septum, indicating that male sexual behaviour is differentially mediated by specific AR populations [86].

In humans, testosterone triggers greater affiliative behaviours in men, e.g., more eye contact, and more self-presentation towards the opposite sex [87]. There is also ample evidence that males with stronger testosterone-controlled signals, such as hair colour [88], facial masculinity [89, 90], and vocalisations [91, 92], are more attractive to females. The emotional mechanisms underlying testosterone-mediated behaviour may be due to its interactions with other neuropeptides, with evidence showing that testosterone upregulates expression of vasopressin and its receptor in brain areas involved in aggressive behaviours including the medial amygdala, lateral hypothalamus, and the medial pre-optic area [84]. Furthermore, testosterone levels are positively correlated with fMRI brain activation in the amygdala of men when viewing fearful or angry faces [93].

In ageing men, progressive testosterone decline is associated with low mood, reduced libido, and poor sexual function [14]. Testosterone insufficiency has also been implicated in sexual dysfunction occurring in post-menopausal women [94]. In hypogonadal men, low sexual interest can be improved with testosterone replacement therapy [95]. However, bringing testosterone into supraphysiological levels confers no additional benefit to sexual activity or sexual interest [96]. Furthermore, a meta-analysis concludes that there is uncertain benefit of testosterone on sexual function in men with normal testosterone levels [97]. In addition, even with adequate testosterone replacement in hypogonadal men, sexual function is not restored to the same level as an age-matched eugonadal male, suggesting that other factors need to be considered [98].

It is known that in women with regular ovulatory cycles, testosterone rises during the follicular phase, reaches maximal levels in the middle third of the cycle, before declining to reach nadir shortly before the next follicular phase [99]. This correlates with measures of female sexual receptivity which are reported to be highest during the follicular phase, when testosterone is maximal [100]. However, the occurrence of other hormonal changes during the mid-cycle potentially modulates the effects of testosterone during this time. Furthermore, interactions between testosterone, oestrogen, and progesterone in women make it difficult to tease out the exact role of testosterone in female sexual behaviour [101]. Despite this, a Cochrane review of 35 trials with a total of 4768 participants concludes that testosterone therapy in post-menopausal women has beneficial effects on sexual function [102] and a trial of testosterone therapy is a recommended management for women with hypoactive sexual desire disorder [103].

### Oestrogen’s role in sex and emotions

Although testosterone plays a fundamental part in orchestrating male sexual behaviours, it is interesting to note that the expression of certain male-typical behaviours requires the aromatisation of testosterone into neuroestrogen [104, 105]. This led to the hypothesis that oestrogen is the main neuropeptide required for male sexual behaviour. In gonadectomised male rats, large doses of oestrogen are able to restore sexual behaviour to the same degree as testosterone replacement [106]. However, when testosterone is compared to oestrogen at physiological doses in gonadectomised male rats, only testosterone is able to restore sexual behaviour [107], confirming that under physiological conditions, testosterone is the principle mediator of male-typical characteristics in rodents.

Sexually receptive behaviours in female rodents are mediated by the ventromedial hypothalamus and the medial amygdala, which are major sites of oestrogen action [108]. Both systemic oestrogen administration and direct injection into the ventromedial hypothalamus induce lordosis behaviour in ovariectomised female rats [108, 109]. In addition, oestrogen-replaced ER knock-out female mice fail to display receptive sexual behaviours in the presence of a male indicating that oestrogen action at its receptor is required for its behavioural effects in female rodents [110].

In humans, there is evidence that raised oestrogen levels prime females to seek out social signals of masculinity. During peak oestrogen levels, women show highest preference for male-specific odours [111], and perceive men with higher testosterone levels [90] and more masculine facial features [112] to be more attractive. Electroencephalogram recordings of women in the ovulatory phase show increased amplitude of the late positive component on viewing sexually arousing images, which indicates deeper emotional processing specific to sexual stimuli during peak oestrogen levels [113].

Thus, in humans, both testosterone and oestrogen have effects on female sexual motivation and behaviours with ongoing debate as to which gonadal steroid is most effective. The current guidelines advocate testosterone for the treatment of sexual desire disorders in women [103] with results from meta analyses concluding that testosterone effectively increases female sexual desire and satisfaction [102, 114]. However, there is also evidence that oestrogen replacement alone, which achieves peri-ovulatory levels, restores sexual desire in hypogonadal women [115]. This suggests that there is considerable overlap in the roles of oestrogen and testosterone in human female sexual function. Oestrogen also has positive effects on mood and potentiates the actions of antidepressants through upregulation of serotonin signalling [116] which may contribute to its effects on sexual desire and satisfaction.

### Summary

Testosterone and oestrogen have well-defined roles in the development and exhibition of sexually driven emotions and behaviours in male and female rodents. This is supported by sex differences in the distribution and density of ERs and ARs within key limbic brain structures. Human studies have found a majority effect for testosterone as a mediator of sexual drive and arousal in men and women; however, questions remain over the role of oestrogen and its interplay with testosterone in the regulation of female sexual behaviours (Fig. 1).

## Oxytocin and vasopressin

Oxytocin and vasopressin are nonapeptide hormones descended from the same ancestral gene; they differ in structure by only two amino acids and are highly conserved among vertebrates [117]. The endocrine effects of oxytocin include induction of uterine contractions during parturition and an essential role in the milk-ejection reflex during lactation [118]. Oxytocin receptors are also found on GnRH neurones [119] and intranasal administration of oxytocin to male rats results in increased GnRH mRNA expression in the anterior hypothalamus [120]. Conversely, vasopressin has an indirect inhibitory effect on GnRH through its potentiation of adrenocortico-tropic hormone (ACTH) [121] and cortisol secretion [122] (Fig. 1). Over the last few decades, research into oxytocin and vasopressin has provided a wealth of information about their roles in mediating social behaviours in many species.

### Distribution of oxytocin and vasopressin in the limbic system

Oxytocin and vasopressin are synthesised in the magnocellular neurones within the paraventricular nucleus (PVN) and supraoptic nucleus (SON), which extend from the hypothalamus to the posterior pituitary where they are stored prior to peripheral release [123]. Central distribution of these peptides occurs in two ways, a small population of parvocellular neurones carry oxytocin and vasopressin directly to target brain regions including the amygdala, hippocampus, olfactory bulbs, and striatum [123]. Secondly, dendritic release of oxytocin and vasopressin into the extracellular space results in diffusion through the brain to distant targets, e.g., from the PVN to the central amygdala (CeA) [124]. In this way, oxytocin and vasopressin are able to exert rapid effects on distant brain areas including important limbic regions.

In rodents, oxytocin and vasopressin are co-expressed in several limbic areas including the medial amygdala, nucleus accumbens, the BNST, and olfactory areas [125]. There appears to be no gross difference in oxytocin distribution between the brains of males and females, whereas vasopressin expression in extra-hypothalamic areas is sexually dimorphic with males having higher numbers of vasopressin-labelled cells in the BNST and the medial amygdala compared to females [126].

Autoradiographical examination in rodents shows high co-localisation of oxytocin and vasopressin-binding sites in limbic brain regions including the amygdala and BNST [127]. However, the same method in post-mortem human brains found marked differences in vasopressin and oxytocin binding with no oxytocin-binding sites detected in the amygdala, nucleus accumbens, or the olfactory bulb [128]. This method of detecting oxytocin and vasopressin binding in humans and non-human primates has since been disputed due to the relative non-specificity and high cross-reactivity of radiolabelled ligands for the oxytocin and vasopressin receptor [128, 129]. More recently, immunohistochemical methods using a monoclonal antibody for the oxytocin receptor have visualised discrete cell bodies and fibres in the central and basolateral regions of the human amygdala, medial pre-optic area (MPOA), anterior and ventromedial hypothalamus and the olfactory nucleus, but not the hippocampus [130].

### Oxytocin’s role in sex and behaviour

Oxytocin enhances sexual behaviours in male and female rodents. Central administration of oxytocin promotes the lordosis response in female rats [131] facilitates erections [132] and reduces latency to ejaculation in male rats [133]. Oxytocin is also involved in the recognition of social cues in animals and humans. Rodents rely almost exclusively on olfactory signals to initiate social and sexual behaviours, and oxytocin is shown to be necessary for the formation of olfactory memories towards conspecifics i.e., mice lacking the oxytocin gene develop “social amnesia” and are unable to recognise the olfactory signature of a mouse they have previously been in contact with [134].

The monogamous prairie vole provides an excellent model for the study of social bonding. These animals form long-term monogamous relationships and display significantly higher concentrations of oxytocin receptors in their limbic structures (amygdala, BNST, and nucleus accumbens) compared to polygamous voles [135]. Furthermore, when monogamous voles are separated from their partner (but not a sibling), they display increased signs of depression on forced swim testing, which is rescued by infusion of oxytocin into the nucleus accumbens shell [136]. To facilitate these social-bonding behaviours, there is evidence that oxytocin suppresses emotions such as anxiety, fear, and avoidance through actions on discrete GABA-ergic neurones within the central amygdala, an area providing major outputs to the autonomic nervous system [137].

In humans, peripheral oxytocin levels are raised during sexual arousal and copulation in women, and to a lesser degree in men [132]. Recent human data have concentrated on oxytocin’s effects on prosocial behaviours such as trust. A study of men and women who were exposed to a single intranasal dose of oxytocin found increased feelings of trust towards unfamiliar neutral faces [138]. Furthermore, oxytocin administration to healthy heterosexual males increases attraction to the face of their female partner [139] indicating a role for oxytocin in human-pair bonding. Oxytocin has also been shown to increase women’s subjective preference for a more masculinised male face [140]. More masculine characteristics are associated with higher testosterone levels, higher social status, and increased reproductive fitness [88]. However, human behaviours are complex and mostly non-stereotyped; thus, reviews of human oxytocin studies have shown small effect sizes and inconsistencies likely relating to contextual and individual differences across experiments [141, 142].

### Vasopressin’s role in sex and emotion

In keeping with its distribution within the limbic brain, vasopressin’s effects on sexual function are also sexually dimorphic. Vasopressin is essential for male sexual behaviours such as aggression towards competitors and mate guarding. This has been demonstrated in male song sparrows, whereby the use of vasopressin antagonists blocks neural responses related to social stressors [143]. In addition, knocking down vasopressin production in the PVN of the hypothalamus in zebra finches using RNA interference techniques significantly reduces social contact time in males and females. Furthermore, this manipulation increases male aggression towards opposite sex, but not to same-sex finches, and significantly reduces female aggression to both sexes [144]. Alongside this, studies in rodents reveal lower aggression towards intruders in male hamsters treated with a vasopressin antagonist [145]. Vasopressin gene knock-out experiments in rats show reduced aggression towards intruders in lactating females and reproductively naïve males but no effect in reproductively experienced males [146]. Moreover, intracerebroventricular (ICV) administration of vasopressin has an inhibitory effect on lordosis behaviour in female rats, which is blocked by pre-treatment with a vasopressin-binding antibody [147]. This effect on sexual receptivity may be species-specific, as the same inhibitory effect is not seen in female hamsters [148]. Furthermore, both vasopressin [149] and cortisol levels [150] are shown to rise during sexual arousal in human males which may be a driver for the stress response required by males to compete for the opportunity to mate with a female.

Similar to oxytocin, vasopressin is also required for the recognition of social cues, as evidenced by distribution of vasopressin receptors in olfactory centres involved in processing social olfactory signals [127]. Furthermore, ICV administration of a vasopressin receptor antagonist blocks social recognition in male rats [151] in the same manner described above during oxytocin antagonism.

In monogamous species, evidence suggests that vasopressin increases affiliative behaviours. This is demonstrated by administration of vasopressin to male titi monkeys resulting in prolonged time spent with their partner versus an opposite sex stranger [152]. In male prairie voles, infusion of a vasopressin receptor (V1R) antagonist into the ventral pallidum blocks partner preference formation [153]. In addition, higher vasopressin levels in human couples are associated with increased feelings of attachment towards their spouse and fewer negative marital interactions [154].

### Summary

Oxytocin and vasopressin are intimately linked in terms of their evolution, distribution within the brain, and their effects on social and sexual behaviours. Both peptides are involved in olfactory recognition in rodents with complementary effects on emotions and prosocial behaviours. Oxytocin is involved in the selection of desirable traits in the opposite sex and promotes intimacy and bonding between individuals. Vasopressin has been shown to facilitate male aggression towards competitors and mediate partner preference behaviours in monogamous species. However, its actions can be sexually dimorphic, vary across species, and are likely to be site-specific within the brain. Oxytocin and vasopressin are therefore essential for the formation of successful pair bonds as part of an integrated network of intrinsic links that unite sex, emotion, and reproduction to facilitate species survival (Fig. 1). Future translational studies will be important to assess whether these observations in animals can be further extrapolated to human society and relationships.

## Glucocorticoids and stress

Stress is intimately linked to reproductive function, with both acute and chronic stress being associated with poor reproductive outcomes. The hypothalamic–pituitary–adrenal (HPA) axis governs our innate endocrine response to stressors and in conditions of HPA over- activity such as Cushing’s syndrome; reproductive function is inhibited [155]. Corticosteroid receptors are found throughout the limbic brain in rodents [156] and primates [157]; and there is evidence that the HPA and HPG axis modulate each other at multiple levels.

Starting at the apex of the HPG axis, both kisspeptin and GnRH are associated with anxiolytic effects, which are in keeping with their roles in promoting positive emotions and behaviours related to reproduction. There is evidence that GnRH inhibits the stress response through mechanisms that involve blocking the effects of corticotropin-releasing hormone (CRH) [64]. Kisspeptin has interactions with serotonin, noradrenaline [41], and dopamine pathways [26], but peripheral kisspeptin administration does not appear to influence the HPA axis in rodents [158] or humans [36]. Corticosterone administration leads to increased GnIH mRNA expression in the Japanese quail [159]. Acute and chronic stress in adult male rats have a similar effect on GnIH expression and are associated with reduction in plasma LH in addition to inhibited sexual behaviour [160]. Furthermore, co-expression of glucocorticoid receptors is seen on 53% of GnIH cells indicating regulation of the HPG axis by glucocorticoids can occur directly at the level of GnIH [160]. Interestingly, a reciprocal relationship is also present, whereby sexual experience can reduce stress-mediated glucocorticoid release [161]. Interactions between the HPG and HPA axis are also seen at the level of ARs. Repeated treatment with testosterone produces anxiolytic effects in castrated male rats [162], and use of the AR antagonist, flutamide not only blocks the anxiolytic effects of testosterone [162] but also further increases anxiety-like behaviour in intact male rats [163].

The behavioural effects mediated by oxytocin and vasopressin are also closely related to their interaction with the HPA axis. Oxytocin has been shown to suppress sympatho-adrenal activity [15] and in socially naïve female prairie voles, cohabitation with a potential male partner activates oxytocin release with associated significant reduction in corticosterone levels [164]. The effect of vasopressin on male and female sexual behaviours can also be partially explained by its role in the HPA axis. Vasopressin potentiates ACTH and cortisol secretion [121, 122], which may contribute to inhibited female sexual behaviour through inhibition of the HPG axis [139, 140]. Conversely, the stimulatory effects of vasopressin on cortisol release are advantageous in males as aggression during mating allows for selection of males with higher reproductive and social status to father offspring, thus improving chances of species survival [88, 165].

### Summary

Stress plays a role in many aspects of sex, emotion, and reproduction. The intrinsic links underlying these interactions are endocrine in nature and involve reciprocal modulation of the HPA and HPG axis at multiple levels.

## Conclusion and future perspectives

As a relatively young discipline, concepts within endocrinology continue to evolve at a rapid pace with a widening body of research in support of complex and far-reaching interactions between endocrine axes and other essential bodily processes. In particular, there has been intense interest in the interface between neuroendocrinology and limbic brain processing with evidence to suggest that the integral links between sexual, emotional, and reproductive brain processes are endocrine in nature. At the apex of the reproductive axis, kisspeptin represents a key link between sex, emotion, and reproduction. Kisspeptin’s actions within the limbic brain include stimulation of the lordosis reflex [30] and induction of erections [37] which both facilitate sex and reproduction. Kisspeptin also integrates emotions with sex and reproduction through its effects on fear [38], mood [41], and sexual arousal [36]. Downstream to kisspeptin, other hormones within the HPG axis are also expressed throughout the limbic system and can modulate limbic brain processing, which suggests that certain emotions and reproductive behaviours are likely to be hardwired through the activation of the HPG axis.

There is also evidence that the HPG axis works in concert with other endocrine mediators including cortisol, oxytocin, and vasopressin to integrate physiological reproductive processes with essential emotions and sexual behaviours required for successful mating.

Looking to the future, we are only just beginning to appreciate the outline of these fundamental neuroendocrine frameworks that integrate sex, emotion, and reproduction. A key area of study will be in unravelling the pathways and mechanisms that underlie these interactions between physiology, psychology, and endocrinology. Translational aspects are also important to explore as animal models are limited by the complexity of human sexual behaviour which is more dependent on learning and context rather than stereotyped actions [15]. From a therapeutic perspective, psychosexual dysfunction affects up to a third of the population [166] and is associated with poor interpersonal relationships and reduced quality of life [167]. Current therapies are very limited, and understanding these intrinsic links between sex, emotion, and reproduction will be key to developing effective therapies in the future.
